# Median sternotomy versus minimally invasive thymectomy for early-stage thymoma: A systematic review and meta-analysis protocol

**DOI:** 10.1097/MD.0000000000018359

**Published:** 2019-12-20

**Authors:** Chai Tianci, Zhimin Shen, Sui Chen, Yuhan Lin, Lei Gao, Zhenyang Zhang, Mingqiang Kang, Jiangbo Lin

**Affiliations:** a Department of Thoracic Surgery, Fujian Medical University Union Hospital; b School of Stomatology, Fujian Medical University, Fuzhou, China.

**Keywords:** median sternotomy, robot-assisted thoracoscopic technique, thymoma, video assisted thoracic technique

## Abstract

**Background:**

The completeness of resection is an important prognostic factor for early resectable thymoma. Since its inception 2 decades ago, median sternotomy has been recognized as the gold standard method for the treatment of all types and stages of thyomas. Minimally invasive surgical techniques, including video-assisted and robot-assisted surgery, have been rapidly developed as an alternative to traditional open approach surgery. Compared with traditional open approach surgery, minimally invasive approach has better cosmetic effect, faster improvement of lung function, reduction of surgical trauma, length of stay, and complications. We believe that this is an appropriate time and there is a need for a systematic, comprehensive, and objective assessment of the 2 surgical modalities in order to provide reliable evidence for clinicians to determine the best treatment for patients with early resectable thymoma.

**Methods:**

Pubmed (Medline), Web of Science, Embase, Cochrane Central Register of Controlled Trials, and Google Scholar will be searched for relevant randomized controlled trials (RCTs), quasi-RCTs, and Hi-Q (high quality) prospective cohort trials published or unpublished in any language before March 1, 2020. Subgroup analysis will be performed in tumor pathological stage and ethnicity. PROSPERO registration number: CRD42019133724.

**Results:**

The results of this study will be published in a peer-reviewed journal.

**Conclusion:**

This study will be the first to assess the efficacy and safety of median sternotomy recognized as the gold standard method for the treatment of all types and stages of thyomas and minimally invasive thymectomy for patients with early-stage thymoma. This study will assess whether minimally invasive thoracoscopic and robotic assisted thymectomy can be used as an alternative to traditional median sternotomy for patients with early resectable thymoma and provide high-quality and reliable evidence for clinicians’ decision-making.

## 1 Introduction

Thymoma is a rare primary intrathymic tumor.^[1]^ Patients with thymoma often have or do not have symptoms of myasthenia gravis.^[2]^ Surgical intervention is the most critical treatment for thymoma and an important prognostic factor for patients.^[3]^

Traditionally, median sternotomy was performed first, then thymic tumors were completely removed, followed by the removal of adipose tissue around thymus and pericardium.^[4,5]^ The completeness of resection is an important prognostic factor for early resectable thymoma.^[3]^ Since its inception 2 decades ago, median sternotomy has been recognized as the gold standard method for the treatment of all types and stages of thyomas.^[4–7]^

Minimally invasive surgical techniques, including video-assisted and robot-assisted surgery, have been rapidly developed as an alternative to traditional open approach surgery.^[8]^ Compared with traditional open approach surgery, minimally invasive approach has better cosmetic effect, faster improvement of lung function, reduction of surgical trauma, length of stay, and complications.^[9–16]^ For the early resectable thymoma, minimally invasive approach is widely used worldwide and has become the preferred surgical approach in some medical centers. We believe that this is an appropriate time and there is a need for a systematic, comprehensive, and objective assessment of the 2 surgical modalities in order to provide reliable evidence for clinicians to determine the best treatment for patients with early resectable thymoma.

## 2 Objective

We will conduct a systematic review and meta-analysis to estimate the efficacy and safety of median sternotomy versus minimally invasive thymectomy for patients with early-stage thymoma.

## 3 Methods

This protocol is conducted according to the Preferred Reporting Items for Systematic Review and Meta-Analysis Protocols (PRISMA-P) Statement.^[17]^ We will report the results of this study adhere to the Preferred Reporting Items for Systematic Reviews and Meta-Analyse (PRISMA) guidelines.^[18]^

### 3.1 Patient and public involvement:

This study will be based on published or unpublished studies and records and will not involve patients or the public directly.

### 3.2 Eligibility criteria

#### 3.2.1 Types of studies

Randomized controlled trials (RCTs) or quasi-RCTs, and high-quality prospective cohort studies published or unpublished will be included, which must have been completed and compared the efficacy and safety of median sternotomy versus minimally invasive thymectomy for patients with early-stage thymoma.

#### 3.2.2 Types of participants

The participants will be adults diagnosed with locally resectable thymoma histologically or cytologically confirmed who were treated with median sternotomy or minimally invasive thymectomy. No restrictions on sex, ethnicity, economic status, and education will be applied.

#### 3.2.3 Types of interventions

All types of median sternotomy versus minimally invasive thymectomy for patients with early-stage thymoma.

#### 3.2.4 Types of outcome measures

##### 3.2.4.1 Primary outcomes

The primary outcome will be overall survival of patients with resectable early-stage thymoma.

##### 3.2.4.2 Secondary outcomes

We will assess the 5-year survival, median survival, recurrence-free survival, complications, length of stay, and quality of life of patients with resectable early-stage thymoma after surgery.

### 3.3 Information sources

We will search PubMed (Medline), Embase, Web of Science, Cancerlit, Google Scholar, and the Cochrane Central Register of Controlled Trials for related studies published before March 1, 2020 without any language restrictions.

### 3.4 Search strategy

We will use the corresponding keywords or subject terms adhered to Medical Subject Heading (MeSH) terms to search for eligible trials in the databases which were mentioned above without any language restrictions.

The PubMed search strategies are shown in Table 1.

**Table 1:**
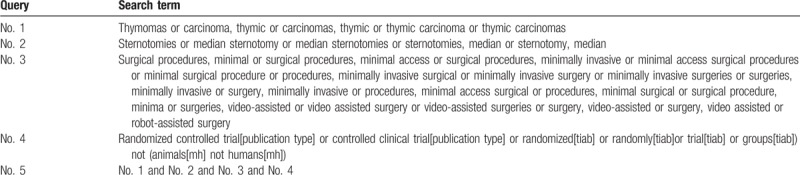
PubMed search strategies.

### 3.5 Data collection and analysis

We will adopt the measures described in the Cochrane Handbook for Systematic Reviews of Interventions to pool the evidence.^[19]^

#### 3.5.1 Study selection

Two reviewers (LG, CTC) will investigate each title and abstract of all literatures searched independently and identify whether the trials meet the inclusion criteria as designed and described in this protocol. Two authors (LG, CTC) will in duplicate and independently screen the full text of all potential eligible studies to exclude irrelevant studies or determine eligibility. The 2 reviewers will list all the studies included and document the primary reasons of exclusion for studies that do not conform to the inclusion criteria. Disagreements between the 2 authors will be resolved by discussing with the third author (SC), if necessary, consulting with the fourth author (MQK). We will show the selection process in details in the PRISMA flow chart.

#### 3.5.2 Data extraction and management

The 2 authors (LG, CTC) will extract the following data independently from the studies included.

- Study characteristics and methodology: country, the first author, publication date, study design, randomization, periods of data collection, total duration of study, follow-up duration, and withdrawals, etc.
- Participant characteristics: age, sex, ethnicity, pathology diagnosis, tumor stage, pathologic tumor size, performance status, and inclusion criteria, etc.
- Interventions: type of operation, number of lymph nodes retrieved, extent of resection, duration of operation, bleeding, postoperative adjuvant therapy, etc.
- Outcome and other data: overall survival, 5-year survival, disease-free survival, median survival, recurrence-free survival, recurrence time, length of stay, length of ICU stay, quality of life, complications, and adverse events, etc.

We will record all the date extracted in a pre-designed table and consult the first author of the trial by e-mail before determining eligibility, if the reported data of which are unclear or missing.

### 3.6 Assessment of risk of bias

Two authors (LG, CTC) will use the Cochrane Handbook for Systematic Reviews of Interventions to assess the risk of bias of each study included independently based on the following ranges: random sequence generation (selection bias); allocation concealment (selection bias); blinding of participants and personnel (performance bias); blinding of outcome assessment (detection bias); incomplete outcome data (attrition bias); selective outcome reporting (reporting bias); other bias.^[20]^ Each domain will be assessed as high, low, or uncertain risk of bias. The results and details of assessment will be reported on the risk of bias graph. EPOC guidelines will be used to assess the risks of non-randomized controlled trials included.^[21]^

### 3.7 Data analysis

The data extracted from the included studies will be synthesised by Review Manager and Stata software. We will conduct a systematic review and meta-analysis only if the data gathered from included trials are judged to be similar enough to ensure a result that is meaningful. The Chi^2^ test and *I*^2^ statistic will be used to assess statistical heterogeneity among the trials included in matched pairs comparison for standard meta-analysis. The DerSimonian and Laird random effect model random effect model will be applied to analyze the data, if there is substantial heterogeneity (*P* < .1 or *I*^2^ statistic >50%) and the trials will be regarded to be obvious heterogeneous. Otherwise, we will adopt fixed effect model to analyze the data. Mantel-Haenszel method will be adopted to pool of the binary data. The results will be reported in the form of relative risk (RR) with 95% confidence interval (CI) of the date. The continuous data will be pooled by inverse variance analysis method and the results will be shown in the form of standardized mean difference (SMD) with 95% confidence interval (CI) of the date.

#### 3.7.1 Subgroup analysis

If there is high heterogeneity and the data are sufficient, subgroup analysis will be conducted to search potential causes of heterogeneity.

Subgroup analysis will be performed in ethnicity, tumor stage, and type of operation.

#### 3.7.2 Sensitivity analysis

Sensitivity analysis will be conducted to assess the reliability and robustness of the aggregation results via eliminating trials with high bias risk. If reporting bias exists, we will use the methods of fill and trim to analyze publication bias.^[22]^

### 3.8 Publication bias

If there are 10 or >10 trials included, we will construct a funnel plot and use Egger test to assess publication bias. If reporting bias is suspected, we will consult the corresponding author via email to determine whether there is reporting bias.

### 3.9 Evidence evaluation

We will evaluate all the evidence according to the criteria of GRADE (imprecision, study limitations, publication bias, consistency of effect, and indirectness bias). The quality of all evidence will be evaluated as 4 levels (high, moderate, low, and very low).^[23]^

## 4 Discussion

Thymoma is a rare intrathymic tumor, but thymoma is the most common primary tumor in the anterior mediastinum of adults. Most patients with thymoma have myasthenia gravis symptoms.^[1,2]^ Thymectomy is a reliable treatment for myasthenia gravis and benign or early thymic tumors. The excision of thymectomy, thymic cavity, and pericardial adipose tissue is a key factor in the prognosis of patients.^[3]^

Traditionally, median sternotomy was performed first, then thorough thymectomy was performed, followed by thymectomy and adipose tissue around the pericardium. Traditional open thymectomy is regarded as the gold standard treatment for thymoma patients.^[4,5]^ However, with the progress of optical and computer-assisted technology, minimally invasive video-assisted thymectomy is becoming more and more popular. The introduction of robotic assistant technology further improves the accuracy and advantages in technical skills and safety.^[8]^

As a substitute for traditional open approach, minimally invasive approach has the advantages of better cosmetic effect, faster improvement of lung function, less surgical trauma, shorter hospital stay, and fewer complications.^[9–14]^ Although minimally invasive thymectomy has many advantages, there is still controversy about which of the 2 surgical techniques has more advantages. We believe this is an opportune time and a systematic, comprehensive, and objective assessment of the 2 surgical procedures is needed to provide reliable evidence for clinicians to determine the optimal treatment for early resectable thymoma.

## Author contributions

**Conceptualization:** Chai Tianci, Zhimin Shen, Sui Chen, Lei Gao.

**Data curation:** Chai Tianci, Zhimin Shen, Lei Gao.

**Formal analysis:** Chai Tianci, Zhimin Shen.

**Funding acquisition:** Lei Gao, Mingqiang Kang, Jiangbo Lin.

**Investigation:** Chai Tianci, Zhimin Shen, Yuhan Lin, Lei Gao, Mingqiang Kang, Jiangbo Lin.

**Methodology:** Chai Tianci, Zhimin Shen, Sui Chen, Zhenyang Zhang.

**Project administration:** Lei Gao, Jiangbo Lin.

**Resources:** Chai Tianci, Zhimin Shen, Yuhan Lin, Lei Gao, Mingqiang Kang.

**Software:** Chai Tianci, Zhimin Shen.

**Supervision:** Lei Gao.

**Validation:** Chai Tianci, Zhimin Shen, Sui Chen, Zhenyang Zhang.

**Visualization:** Zhimin Shen.

**Writing – original draft:** Chai Tianci, Lei Gao.

**Writing – review & editing:** Lei Gao.
